# Defensin-driven viral evolution

**DOI:** 10.1371/journal.ppat.1009018

**Published:** 2020-11-24

**Authors:** Karina Diaz, Ciara T. Hu, Youngmee Sul, Beth A. Bromme, Nicolle D. Myers, Ksenia V. Skorohodova, Anshu P. Gounder, Jason G. Smith

**Affiliations:** Department of Microbiology, University of Washington School of Medicine, Seattle, Washington, United States of America; University of Michigan, USA, UNITED STATES

## Abstract

Enteric alpha-defensins are potent effectors of innate immunity that are abundantly expressed in the small intestine. Certain enteric bacteria and viruses are resistant to defensins and even appropriate them to enhance infection despite neutralization of closely related microbes. We therefore hypothesized that defensins impose selective pressure during fecal-oral transmission. Upon passaging a defensin-sensitive serotype of adenovirus in the presence of a human defensin, mutations in the major capsid protein hexon accumulated. In contrast, prior studies identified the vertex proteins as important determinants of defensin antiviral activity. Infection and biochemical assays suggest that a balance between increased cell binding and a downstream block in intracellular trafficking mediated by defensin interactions with all of the major capsid proteins dictates the outcome of infection. These results extensively revise our understanding of the interplay between defensins and non-enveloped viruses. Furthermore, they provide a feasible rationale for defensins shaping viral evolution, resulting in differences in infection phenotypes of closely related viruses.

## Author summary

Defensins are potent antimicrobial peptides that are found on human mucosal surfaces and can directly neutralize viruses. They are abundant in the small intestine, which is constantly challenged by ingested viral pathogens. Interestingly, non-enveloped viruses, such as adenovirus, that infect the gastrointestinal system are unaffected by defensins or can even appropriate defensins to enhance their infection. In contrast, respiratory adenoviruses are neutralized by the same defensins. How enteric viruses overcome defensin neutralization is not well understood. Our studies are the first to show that defensins can drive the evolution of non-enveloped viruses. Furthermore, we identify important components within human adenovirus that dictate sensitivity to defensins. This new insight into defensin-virus interactions informs our understanding of mucosal immunity to viral infections.

## Introduction

Defensins are small, cationic, and amphipathic peptides that constitute a conserved component of the innate immune system [1,2]. Expression of these host defense peptides by humans, despite the presence of a refined adaptive immune system, highlights their key role in protection from microbes. Although there are examples of both α- and β-defensins with antibacterial activity and antiviral activity against enveloped viruses, only α-defensins affect the infectivity of non-enveloped viruses [1,3,4]. There are two major classes of α-defensins: myeloid, which are produced by neutrophils, and enteric, which are secreted in the genitourinary tract and by Paneth cells in the crypts of the small intestine. Human defensin 5 (HD5) is the most abundantly expressed human enteric α-defensin and has the greatest inhibitory activity against human non-enveloped viruses, including polyomaviruses, papillomaviruses, and some serotypes of adenovirus (AdV) [5–12]. Although differing in some respects between viruses, a conserved mechanism by which non-enveloped viruses are neutralized by α-defensins has emerged. In essence, stabilization of the capsid by α-defensin binding leads to changes in uncoating and intracellular trafficking, thereby preventing the genome from reaching the nucleus to initiate replication [1,5,6,8–11,13–15].

Despite their broad antimicrobial activity, enteric α-defensins are not able to inhibit all non-enveloped viruses. Echovirus, reovirus, and enteric AdVs from both humans and mice are resistant to enteric α-defensin inhibition [9,16–18]. One hypothesis to explain these observations is that resistance stems from evolutionary pressure imposed by enteric α-defensins during fecal-oral transmission. Consistent with this hypothesis, rather than kill the enteric bacterial pathogen shigella, HD5 was recently found to promote its cell binding and infection [19,20]. Moreover, enteric α-defensins play a role in shaping the microbial communities of the gastrointestinal tract through differential susceptibility of commensal bacteria to defensin killing [21–23]. Collectively, these observations suggest that defensin-driven evolution of enteric microbes is a common cross-kingdom occurrence.

To directly test the ability of enteric α-defensins to drive viral evolution, we used human AdV (HAdV). We previously demonstrated that HAdV infection can either be neutralized by, resistant to, or enhanced by HD5, depending on serotype [9]. Thus, the naturally occurring diversity of HAdVs is an appealing substrate to identify viral determinants for neutralization and enhancement by defensins. The icosahedral AdV capsid consists of three major proteins: hexon, penton base (PB), and fiber. In previous studies, we used a rational design approach to identify the vertex proteins, fiber and PB, as determinants of HD5 neutralization [9]. Here, we evolved HD5 resistance in a defensin-sensitive serotype, demonstrating the capability of enteric α-defensins to drive viral evolution. From this, we identified a hypervariable loop in hexon as a novel determinant that substantially revises our understanding of the mechanism of HD5 neutralization.

## Results

### Hexon hypervariable region 1 is a novel determinant of HD5 sensitivity

**Fig 1:**
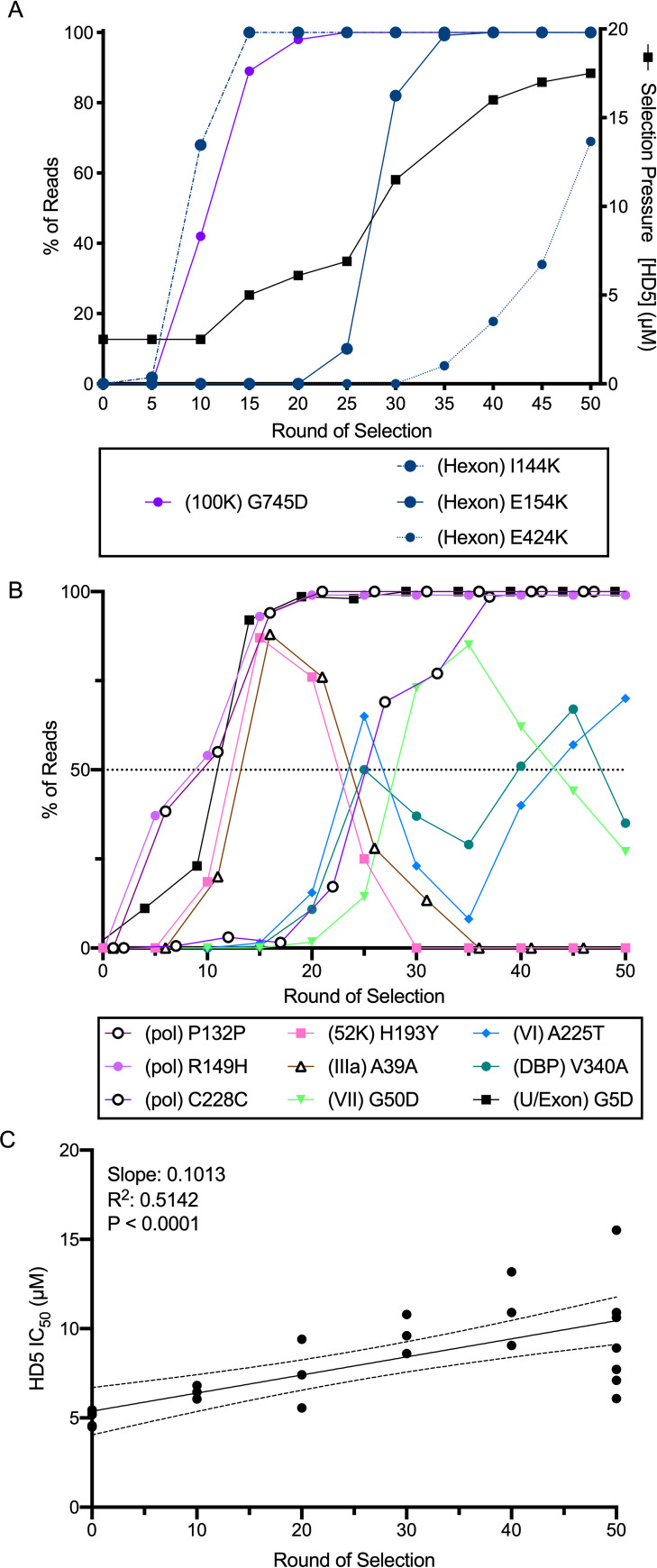
Selection of HD5-resistant HAdV-5. HAdV-5 “mutator” virus was pre-bound to 293β5 cells and then incubated with HD5 to select for resistant viruses. (A) The right y-axis indicates the concentration of HD5 used for selection (solid black line). The left y-axis indicates the percentage of reads containing the denoted mutations in hexon (blue) and L4-100K (purple) in pools of virus expanded from the bulk selected population. (B) All mutations in other proteins that were found in at least 50% of the population during selection. (C) HD5 IC_50_s of pools of virus expanded from the bulk selected population were determined on 293β5 cells. Each point is an independent experiment, and linear regression with 95% confidence bands is graphed.

To determine whether HD5 could impose a selective force for HAdV evolution, we utilized a previously described HAdV-5-based “mutator” vector encoding a polymerase with reduced fidelity to facilitate *de novo* mutagenesis [24]. Over 50 passages, we found that the HD5 IC_90_ used for selection increased 7-fold from ~2.5 μM to ~17.5 μM (black line, Fig 1A). We expanded the viral pool from every 5^th^ round of HD5 selection and the 10^th^ and 20^th^ rounds of passaging control virus. As expected, there was a significant positive correlation between the round of selection and the HD5 IC_50_ of the population (Fig 1C). Whole genome sequencing yielded an average of 6000 mapped reads per base. Both the initial inoculum and the passaged control samples contained only low frequency (<1%) mutations. In contrast, we observed numerous mutations across the viral genome that exceeded 1% frequency in the HD5 selected samples (Table 1). Mutations in the DNA binding protein (V340A), VI (A225T), VII (G50D), 52K protein (H193Y), and IIIa (A39) reached a frequency of >50%; however, none of these mutations stayed above this threshold (Fig 1B). In contrast, mutations in polymerase (R149H, P132, and C228), the U Exon (G5D), hexon (I114K and E154K), and L4-100K (G745D) became fixed in the population as early as the 15^th^ round of selection (Fig 1A and 1B). In addition, a third mutation in hexon (E424K) was trending towards fixation (Fig 1A).

**Table 1:** Number of mutations found at >1% frequency at any point during selection with HD5.

Protein	S	NS	Tv	Ts
eGFP	1	5	2	4
Hexon	2	3	1	4
Fiber		2		2
VI	1	1		2
IX	1	1		2
TP	1	2		3
VII	1	2		3
DBP		2		2
100K	1	2		3
33K		1		1
52K	1	1		2
U Exon		1		1
Pol	2	3	1	4
**TOTAL**	**11**	**26**	**4**	**33**

S: Synonymous Mutation

NS: Non-Synonymous Mutation

Tv: Transversion, Ts: Transition

**Fig 2:**
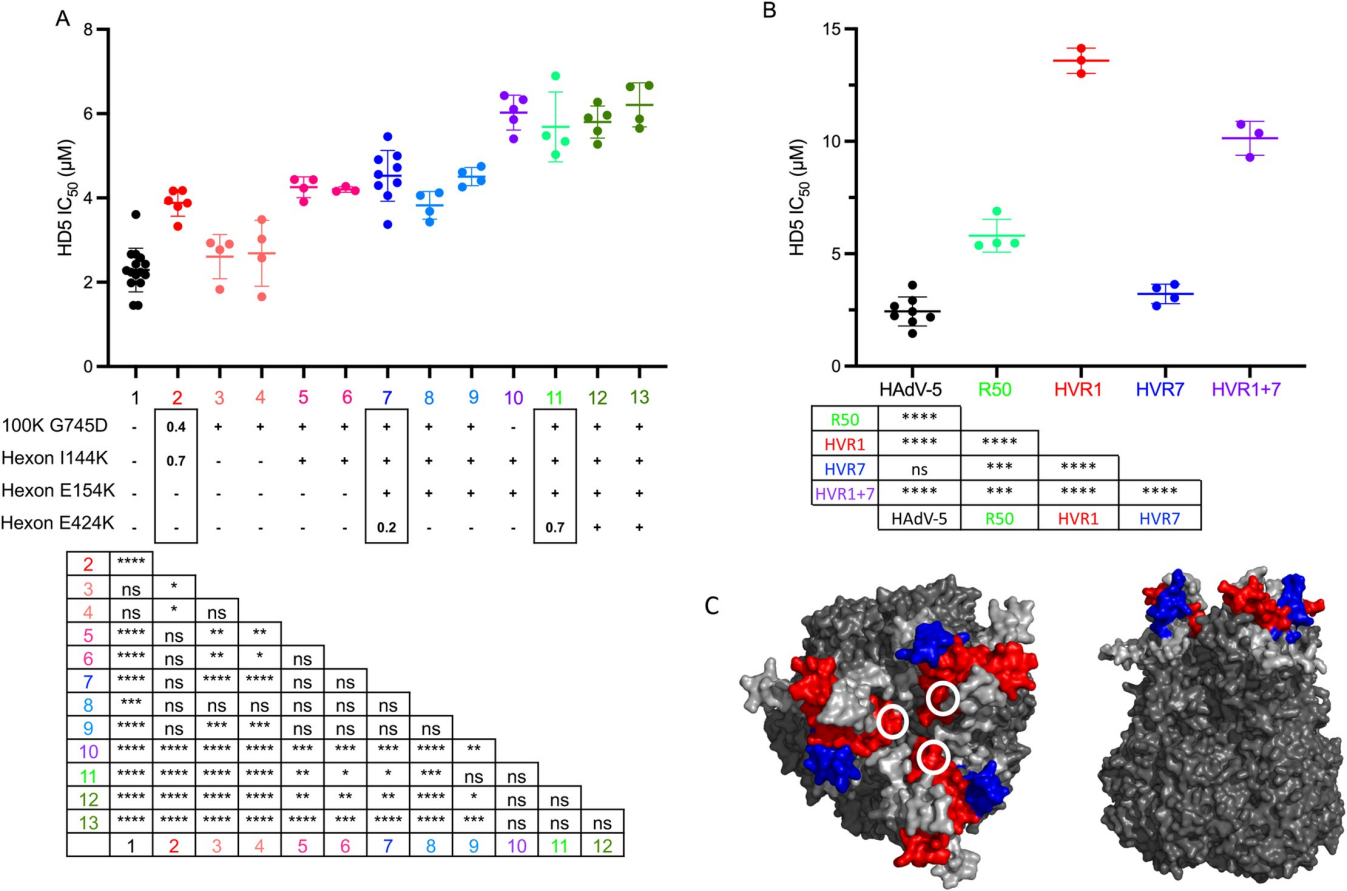
Hexon HVR1 is a determinant of HD5-mediated neutralization. (A) The HD5 IC_50_s of HAdV-5 “mutator” virus (column 1, black); pools of virus expanded from rounds 10 (column 2, red), 40 (column 7, dark blue), and 50 (column 11, light green); plaque purified viruses from rounds 10 (columns 3–4, salmon; columns 5–6, pinks), 40 (columns 8 and 9, blues), and 50 (columns 12 and 13, greens) of the selection; and an engineered virus containing only the indicated hexon mutations (column 10, purple) were determined on 293β5 cells. The presence of each hexon and L4-100K mutation is denoted below the graph. For the pooled viruses, the fraction of reads containing each mutation is indicated within the rectangles. Each point is an independent experiment, and bars are the mean ± SD. The results of one-way ANOVA with Tukey’s multiple comparison test are indicated by asterisks below. (B) The HD5 IC_50_s of the indicated viruses were determined on A549 cells. Each point is an independent experiment, and bars are the mean ± SD. Virus expanded from the 50^th^ round of selection (R50) is equivalent to sample 11 in panel A. Results of one-way ANOVA with Tukey’s multiple comparisons test are shown by asterisks below. (C) Space-filling model of the structure of a HAdV-5 hexon trimer (PDBID:6CGV [44]) from top and side views. HVR1 (blue) is modeled using the structure of the shorter HVR1 from species D HAdV-26 (PDBID:5TX1 [45]). White circles indicate the position of E424 in HVR7 (red). All other HVRs are light gray.

Each of the hexon mutations introduced positive charge in one of two hypervariable regions (HVRs), HVR1 (I144K and E154K) or HVR7 (E424K), located on the outer face of hexon (Fig 2C); therefore, we focused on their contribution to HD5 resistance. We plaque purified viruses with one, two, or three of these hexon mutations in combination with the L4-100K mutation. We were unable to isolate viruses that contained only hexon mutations in the absence of the L4-100K mutation, perhaps due to the role of this protein as a chaperone for the proper folding, trimerization, and transport of hexon [25,26]. Thus, we engineered a virus containing both HVR1 mutations in the absence of the L4-100K mutation. Viruses that contained only the mutation in L4-100K had an IC_50_ equivalent to that of HAdV-5 (Fig 2A, salmon). Viruses with the L4-100K mutation and only one (pink) or both (blue) of the hexon mutations in HVR1 had a significantly higher IC_50_ compared to the starting population but were equivalent to each other. The presence of all three hexon mutations (green) resulted in a ~3-fold increase in IC_50_ from the starting population. Interestingly, the virus engineered with the two mutations in HVR1 but without the mutation in L4-100K (purple) was just as resistant to HD5 as viruses with all three hexon mutations and L4-100K. Taken together, these results indicate an important role for HVR1 and HVR7 in sensitivity of HAdV-5 to neutralization by HD5.

To further explore the potential importance of HVR1 and HVR7 for HD5 interactions, we created chimeric viruses at these regions between HAdV-5 and HAdV-64, an HD5-enhanced serotype [9]. Although we recovered virus from genomic constructs containing HAdV-64 HVR1 and HVR7 in the HAdV-5 background, we failed to recover the reverse chimeras. Placing HAdV-64 HVR1 into HAdV-5 increased the HD5 IC_50_ of the virus ~5-fold over HAdV-5, which was also significantly higher than the HD5 IC_50_ of the pool of viruses from round 50 of the selection (Fig 2B). In contrast, placing the HAdV-64 HVR7 into HAdV-5 had no effect. Replacing both HVR1 and HVR7 in HAdV-5 resulted in an intermediate phenotype compared to the single HVR changes. Overall, these studies identify HVR1 as a novel determinant for HAdV-5 neutralization by HD5.

### Multiple sequence elements in penton base contribute to HD5 sensitivity

**Fig 3:**
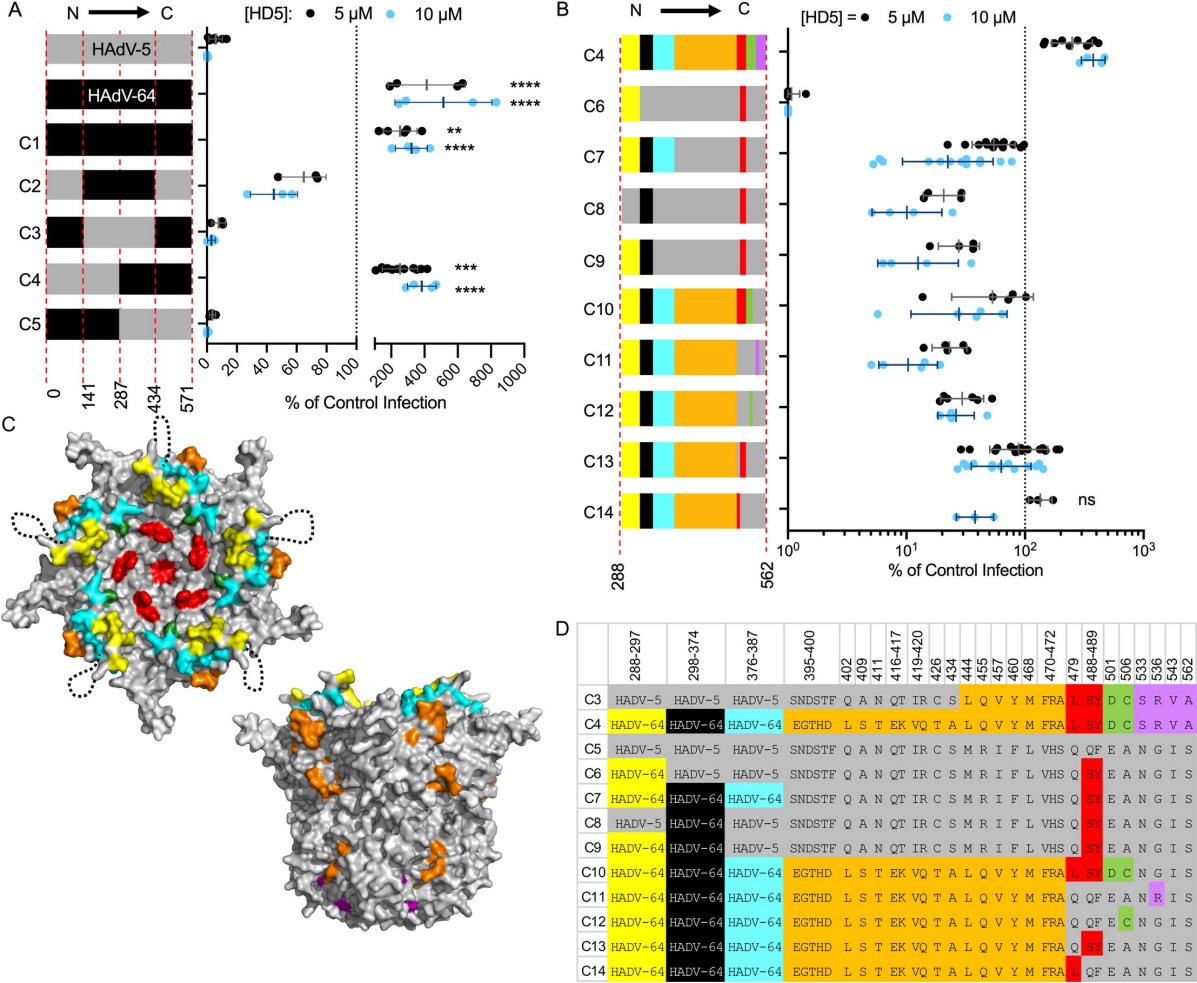
Determinants of HD5-mediated neutralization are located within the C-terminal half of penton base. (A and B) HAdV-5, HAdV-64, and chimeric viruses were incubated with 5 μM (black) or 10 μM (blue) HD5 and then assessed for infectivity on A549 cells. In (A), colors denote sequences derived from HAdV-5 (grey) or HAdV-64 (black), and amino acid residue numbers refer to HAdV-5 PB. In (B), colors correspond to the chart in (D) of the differential amino acid residues between HAdV-5 and HAdV-64 in the C-terminal half of PB, numbered according to HAdV-5. Each point is an independent experiment, and bars are the mean ± SD of the percent infectivity compared to control cells infected with each virus in the absence of HD5. Note that the same data for C4 are plotted on both graphs. For (A), results of two-way ANOVA with Dunnett’s multiple comparison to HAdV-5 are indicated by asterisks. For (B), all comparisons to chimera C4 are significant (P ≤ 0.01) except where indicated as not significant (ns). (C) Space-filling model of the crystal structure of a pentamer of HAdV-5 PB (PDBID:6CGV [44]) in top and side views, with differential residues colored as in (D). The unresolved RGD loop (residues 298–374) is denoted by a dotted black line.

The absence of mutations in fiber and PB in the evolved viral populations was unexpected based on our previous studies [9]. To substantiate our prior findings and more narrowly delineate neutralization determinants in PB, we created a series of HAdV-5-based chimeric viruses in which portions of PB were swapped with the corresponding residues from HAdV-64. These constructs also contain a DTET to GYAR mutation in fiber, which acts in concert with changes in PB to ablate HD5-dependent neutralization [9]. Initially, we chose highly conserved regions spaced evenly within the HAdV-5 PB sequence as junction points for our chimera designs. As in our prior studies [9], HAdV-5 infection was potently neutralized, HAdV-64 infection was enhanced 4- to 5-fold, and infection by a chimera (C1) containing the entire HAdV-64 PB was moderately enhanced 2- to 3-fold when incubated with 5 μM or 10 μM HD5 (Fig 3A). If C-terminal residues 288 to 571 of PB are from HAdV-5 (C5), then the virus is neutralized by HD5. If they are from HAdV-64 (C4), then infection is not neutralized but enhanced. An intermediate phenotype of partial HD5 sensitivity was observed when residues 288 to 434 were derived from HAdV-64 and residues 435 to 571 were derived from HAdV-5 (C2), while the inverse construct (C3) completely recapitulated the HD5-sensitivity of HAdV-5. Thus, residues 288 to 434 from HAdV-5 are necessary for complete neutralization, while residues 435–571 also contribute.

Although not exhaustive, additional chimeras were created to probe the contribution of specific variable sequences within the C-terminal half of PB to HD5-dependent neutralization (Fig 3B and 3D). In the description that follows and in Fig 3B–3D, colors indicate where the equivalent residues from HAdV-64 are substituted into HAdV-5 PB. The highly variable RGD loop (black) from HAdV-5 is necessary for HD5 neutralization. A moderately conserved motif C-terminal to the RGD loop (cyan) also contributes to HD5 sensitivity, while one N-terminal to the RGD loop (yellow) does not (compare C6, C7, C8, and C9). And, the four variable positions between residues 533 and 562 (purple) contribute to neutralization (compare C4 and C10), while the 22 variable residues between 395 and 472 (orange) do not (compare C7 and C13). Although no definitive conclusions can be drawn, a comparison of C10 and C11 suggests that the variable positions between residues 479 and 506 (red and green) also contribute modestly to neutralization. Collectively, this analysis suggests that complete neutralization of HAdV-5 by HD5 reflects the additive effects of multiple residues in PB.

### The role of capsid proteins in determining the outcome of HD5-virus interactions depends on the timing of exposure to HD5 relative to cell binding

**Fig 4:**
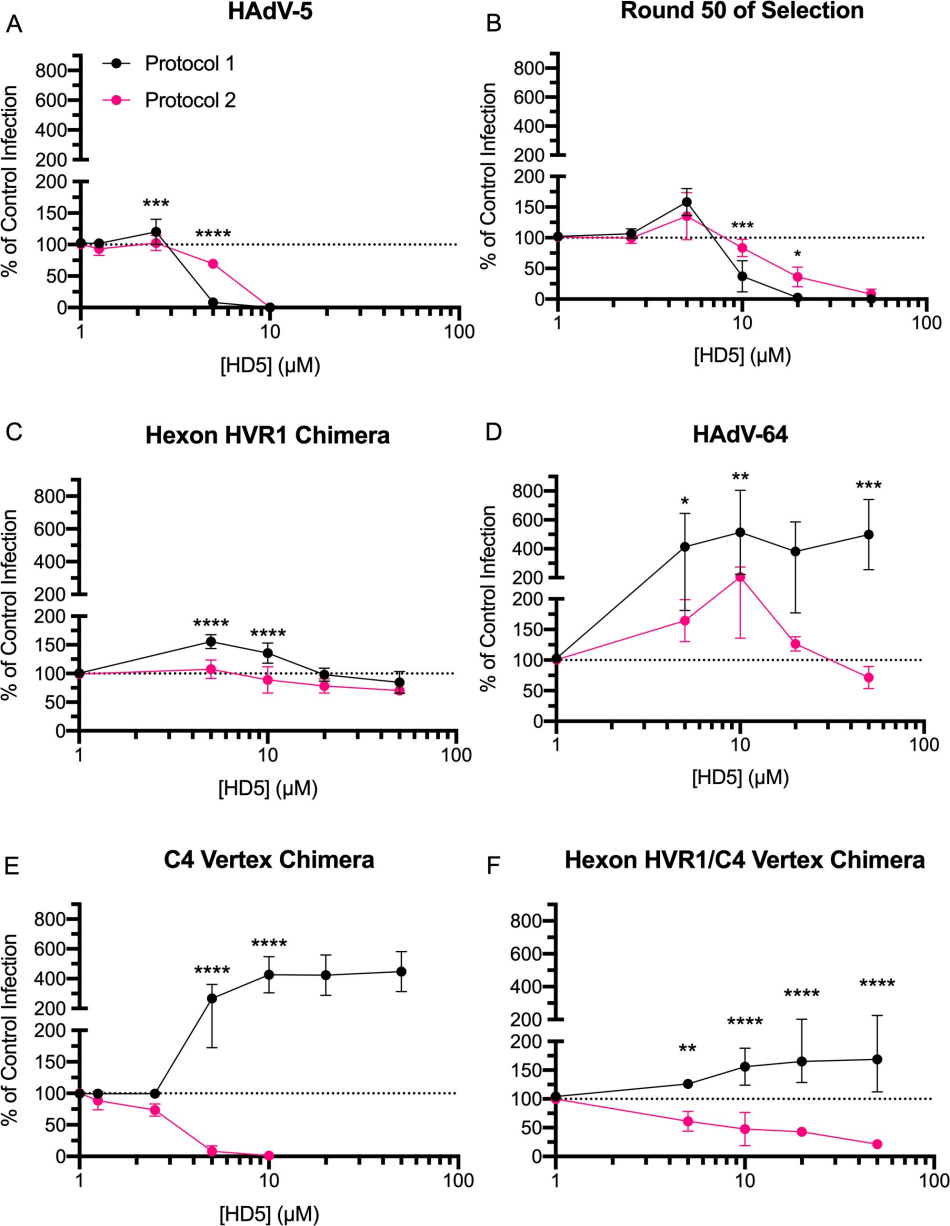
Hexon and vertex play different roles during virus-defensin interactions. Purified (A) HAdV-5, (B) virus expanded from round 50 of selection, (C) hexon HVR1 chimera, (D) HAdV-64, (E) C4 vertex chimera and (F) hexon HVR1/C4 vertex chimera were either incubated with HD5 and then added to A549 cells (protocol 1 –black) or bound to A549 cells prior to HD5 addition (protocol 2 –pink). Data are the mean of 3 to 11 independent experiments ± SD. Results of two-way ANOVA with Sidak’s multiple comparisons at each HD5 concentration are indicated by asterisks.

Rational design and directed evolution implicated different major capsid proteins as HD5 neutralization determinants. We suspected that this discrepancy was protocol dependent. We therefore determined the HD5 IC_50_ for key viruses by either pre-incubating virus with HD5 and then adding this mixture to cells, as in the assessment of PB chimeric viruses (protocol 1), or by adding the defensin to virus pre-bound to cells, as in the selection for HD5-resistance (protocol 2). The phenotypes of HAdV-5 (Fig 4A), the round 50 pool (Fig 4B), and hexon HVR1 chimera (Fig 4C) were largely protocol-independent. The only difference in the phenotype of HAdV-64 (Fig 4D) was enhancement in protocol 1 compared to resistance in protocol 2. In contrast, the phenotype of the C4 vertex chimera was dramatically protocol-dependent (Fig 4E), demonstrating that cell binding can alter HD5 sensitivity.

We then created and tested an additional construct combining the changes from the C4 vertex and hexon HVR1 chimeras. In both protocols, the hexon HVR1/C4 vertex chimera exhibited an intermediate phenotype. In protocol 1, it was resistant to neutralization by HD5 at all concentrations but not enhanced (Fig 4F). In protocol 2, it was ~3-fold more HD5-resistant than the C4 vertex chimera but ~2-fold more HD5-sensitive than the hexon HVR1 chimera. Thus, both hexon HVR1 and the vertex are important determinants of viral infectivity in the presence of HD5, but they appear to have additive rather than synergistic effects.

### Vertex and hexon proteins dictate HD5 binding to the viral capsid

**Fig 5:**
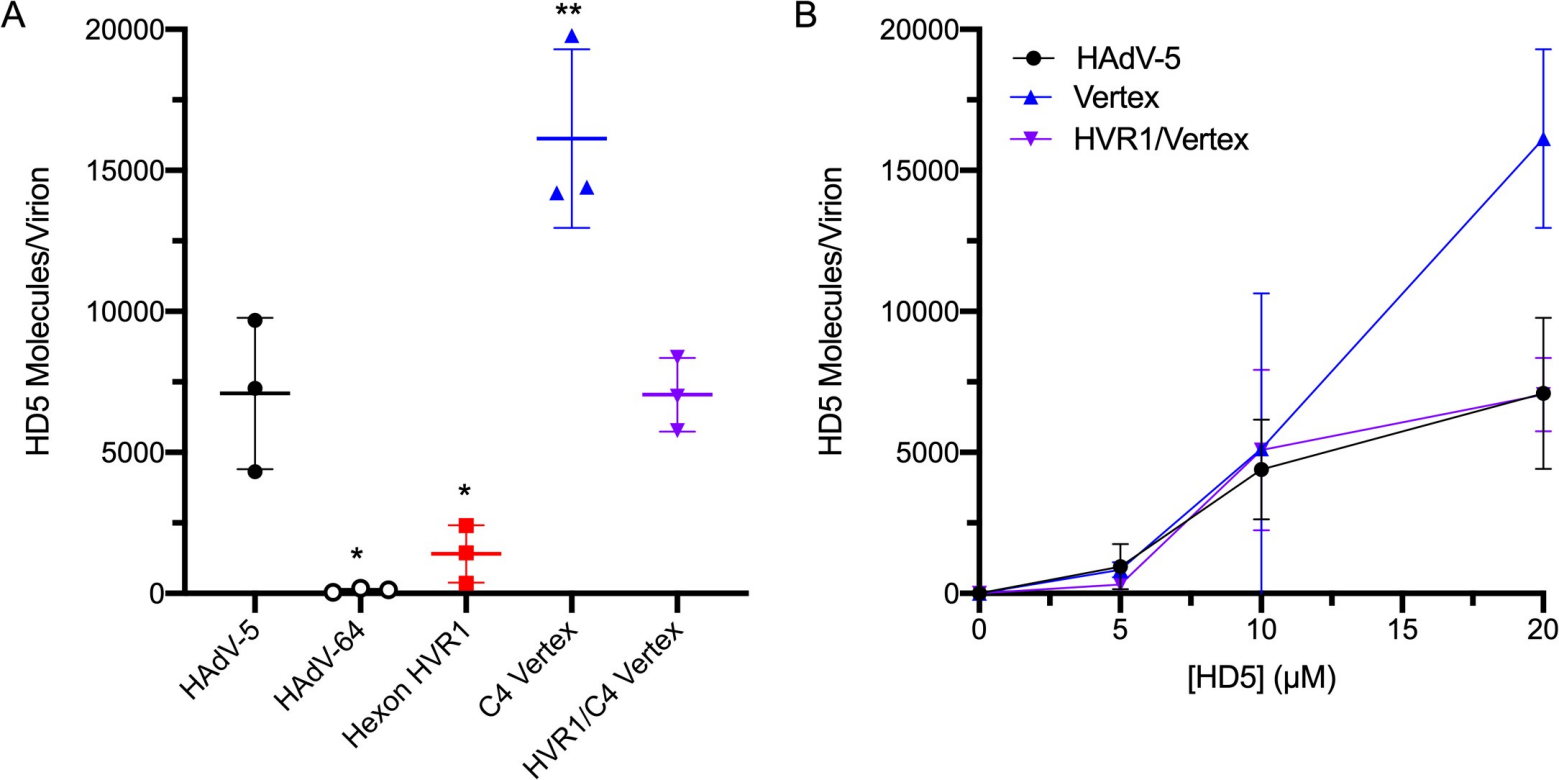
Both hexon HVR1 and vertex contribute to HD5 binding. (A) HAdV-5, HAdV-64, and chimeric viruses were incubated in the presence of 20 μM HD5. The amount of HD5 bound per virion was quantified from 3 independent experiments. (B) HD5 molecules bound per virion of HAdV-5 and chimeras C4 vertex and hexon HVR1/C4 vertex were also determined in the presence of 5 μM and 10 μM HD5. Note that the data for 20 μM HD5 for these viruses in (A) are reproduced in (B). Lines are the mean ± SD. The results of ordinary one-way ANOVA with Dunnett’s multiple comparisons to HAdV-5 is denoted by asterisks.

Based on previous studies demonstrating a direct interaction between HD5 and HAdV [9, 11,12,15], we quantified the average number of HD5 molecules bound to the capsid of each virus. At a concentration of 20 μM, HD5 binds at a high molar ratio to HAdV-5 (7090 +/- 1550 molecules of HD5 per HAdV-5 virion), as shown previously [9], and ~83-fold less to HAdV-64 (Fig 5A). Interestingly, the C4 vertex chimera bound ~2.3-fold more HD5 than HAdV-5 did, while the hexon HVR1 chimera bound ~5-fold less. Finally, HD5 bound to the combined hexon HVR1/C4 vertex chimera at the same levels as HAdV-5. At lower concentrations (5 μM and 10 μM), HD5 binding to HAdV-5, C4 vertex chimera, and hexon HVR1/C4 vertex chimera was equivalent (Fig 5B). Thus, changes in both hexon and the vertex proteins alter the stoichiometry of the HD5-capsid interaction.

### HD5 binding to the viral capsid increases AdV binding to cells

**Fig 6:**
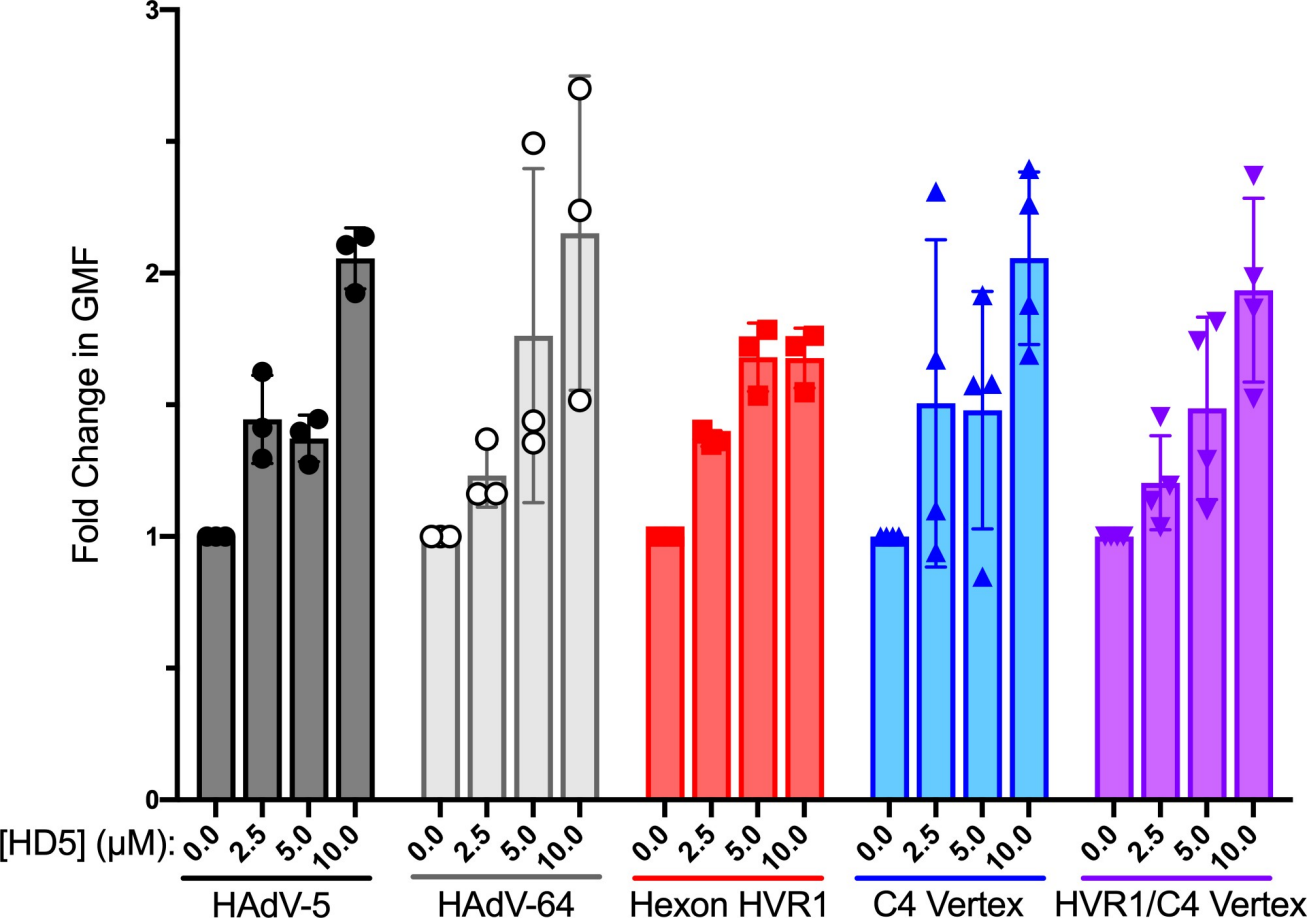
HD5 increases the binding of viruses to cells regardless of infection phenotype. AF488-labeled HAdV-5, HAdV-64, and chimeric viruses were incubated with or without HD5 and then allowed to bind to A549 cells in the cold (as in Fig 4, protocol 1). Data are the fold change in geometric mean fluorescence (GMF) of cells bound by the respective labeled virus incubated with the indicated HD5 concentrations relative to no HD5 for each virus. Each point is an independent experiment, and bars are the mean ± SD of 3–4 independent experiments. Differences between each genotype and HAdV-5 at each HD5 concentration are not significant by ordinary two-way ANOVA with Dunnett’s multiple comparison test.

One possible mechanism for enhanced infection in protocol 1 is an HD5-dependent increase in binding of HAdV to cells, which we have previously demonstrated for HAdV-5 [9,15]. To test this hypothesis, we quantified the amount of Alexa Fluor 488 (AF488)-labeled virus bound to cells after pre-incubation with HD5 (Fig 6). As expected, we observed a 1.4- to 2.1-fold increase in HAdV-5 binding to cells that was HD5 dose-dependent. Despite varying levels of HD5 bound to each genotype (Fig 5A) and regardless of the ultimate effect of HD5 on infection (Fig 4), HD5 enhanced binding of all genotypes to cells to a similar extent. These results are consistent with a model in which HAdV binding to cells is enhanced by HD5, which results in enhanced infection if the virus is not blocked at a downstream step.

### The composition of both hexon HVR1 and the vertex proteins influence fiber stability upon HD5 binding

**Fig 7:**
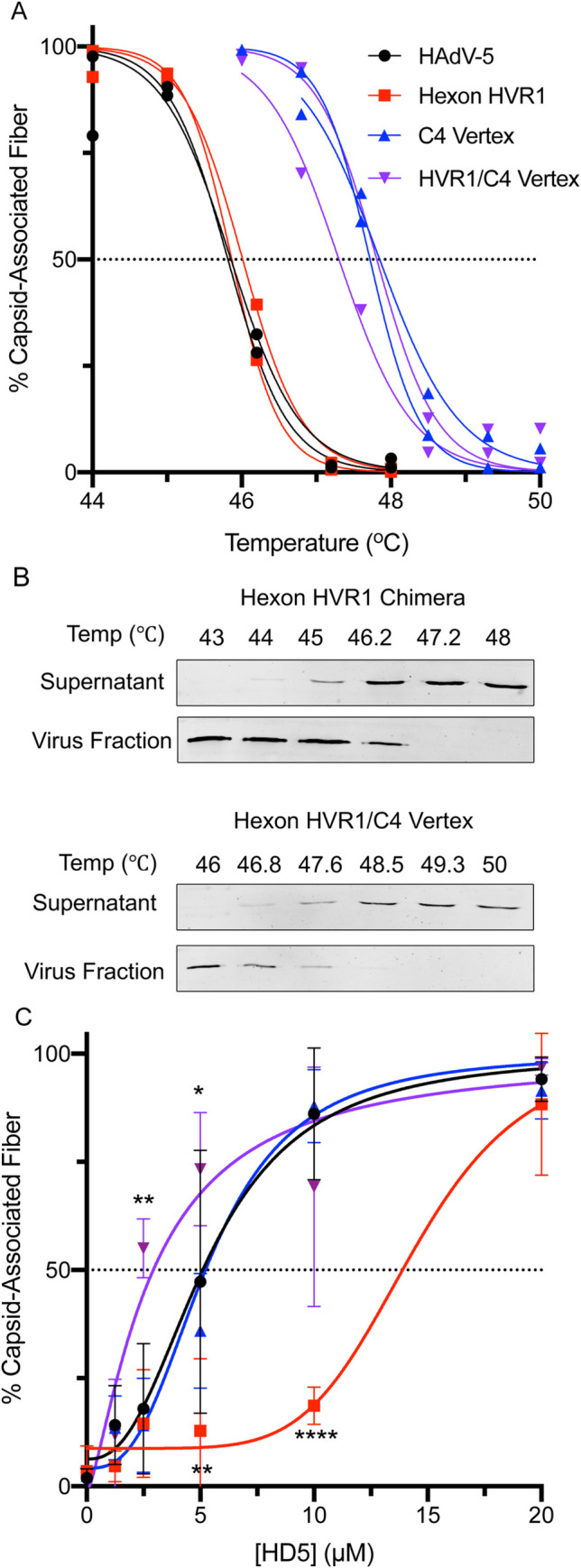
Fiber thermostability does not correlate with infection phenotype. The percent of fiber that remains capsid associated was determined (A) as a function of temperature in the absence of HD5 or (C) as a function of HD5 concentration for HAdV-5 (black) and hexon HVR1 chimera (red) at 48°C and for C4 vertex chimera (blue) and hexon HVR1/C4 vertex chimera (purple) at 49.3°C. (B) Representative immunoblots from the temperature gradients of hexon HVR1 and hexon HVR1/C4 vertex chimeras are shown. In (A) each point and line is an individual replicate. In (C), each point is the mean ± SD of 3 independent experiments, and the results of two-way ANOVA with Dunnett’s multiple comparisons to HAdV-5 are denoted by asterisks.

Our prior studies are consistent with a mechanism in which HD5 neutralizes HAdV-5 by stabilizing the capsid and preventing shedding of the vertex proteins [9,11]. Thus, capsid changes could impact the thermostability of the virus in the presence or absence of HD5. We examined this property at pH 7.4 to approximate the neutral pH of the intestinal lumen where HD5 is most abundantly expressed. We first determined the temperature at which 50% of fiber dissociates from the viral capsid (Tm) in the absence of HD5. As expected, all viruses remained intact at 44°C, and fiber was completely dissociated at 50°C (Fig 7A and 7B). The Tm of the hexon HVR1 chimera was identical to that of HAdV-5 (46°C), while the Tms of the C4 vertex and hexon HVR1/C4 vertex chimeras were similar to each other and warmer than that of HAdV-5 by ~2°C. Thus, amino acid changes in the C4 vertex stabilize the fiber-capsid interaction, while amino acid changes in hexon HVR1 have no effect. We then tested the effect of HD5 on fiber dissociation, when samples were heated to 2°C above the Tm to assure full fiber dissociation in the absence of HD5. Despite their different infection phenotypes and HD5 binding capacities, the fiber of each of these viruses was fully capsid-associated upon incubation with 20 μM HD5 (Fig 7C). HAdV-5 and C4 vertex chimera fibers were 50% capsid-associated at 5 μM HD5 and had identical HD5-dependent dissociation profiles. The hexon HVR1/C4 vertex chimera required a 2-fold lower HD5 concentration than HAdV-5 to be 50% capsid-associated and was significantly more stabilized than HAdV-5 at both 2.5 μM and 5 μM HD5. In contrast, the hexon HVR1 chimera required at least a 2-fold higher HD5 concentration than HAdV-5 to be 50% stabilized. Overall, the composition of both HVR1 and the vertex influence HD5-mediated fiber stabilization; however, the phenotype of the combination does not mirror the individual contributions of each capsid component.

### Neutralization correlates with altered intracellular trafficking

**Fig 8:**
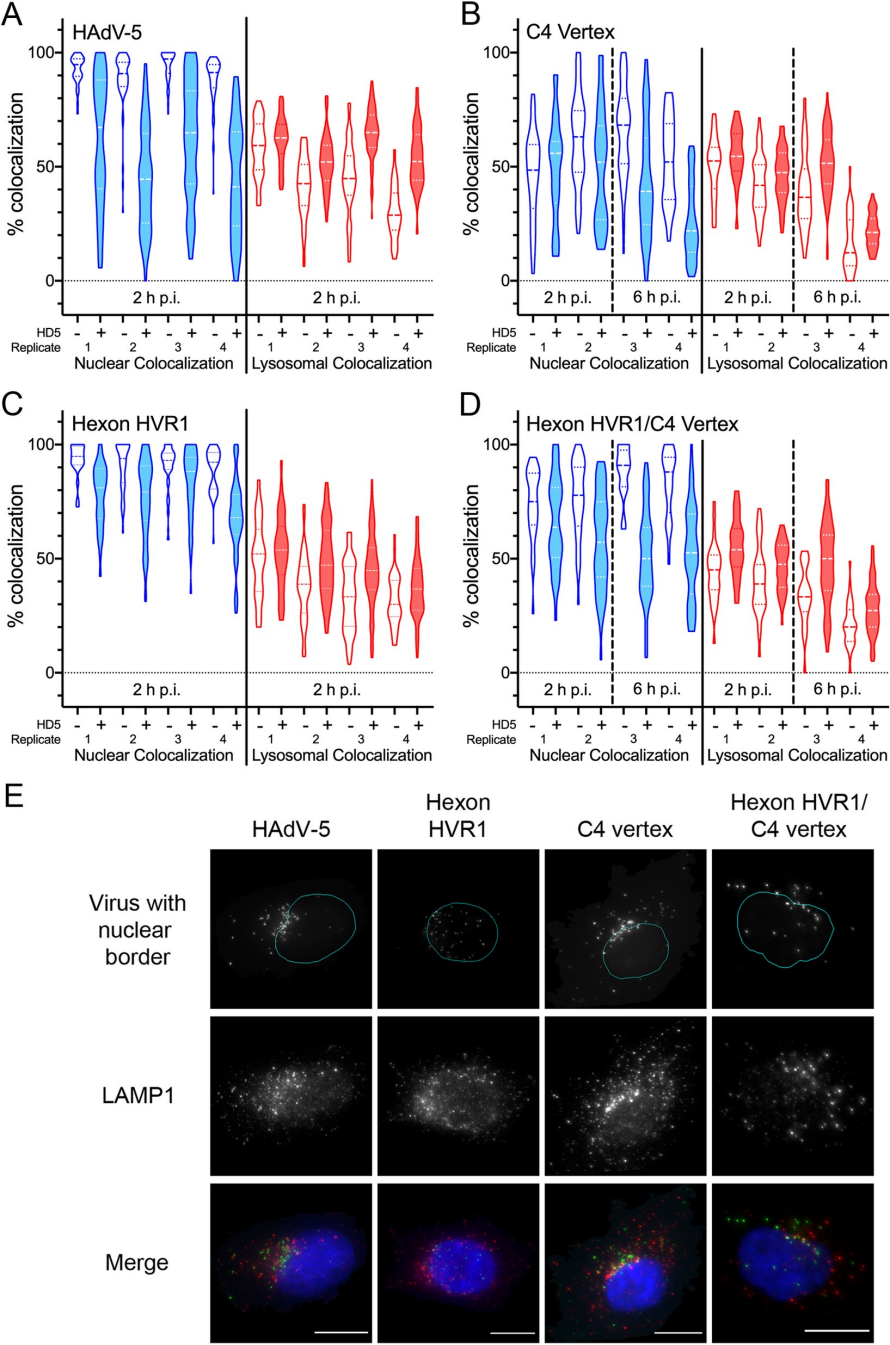
HD5 neutralization correlates with a reduction in nuclear localization. AF488-labeled HAdV-5 (A), C4 vertex (B), hexon HVR1 (C), and hexon HVR1/C4 vertex (D) were bound to A549 cells in the cold and then incubated with (shaded plots) or without (unshaded plots) 10 μM HD5 (as in Fig 4, protocol 2). Cells were then warmed to 37°C and fixed at the indicated times post-infection (p.i.). Images obtained by epifluorescence microscopy were analyzed for percent colocalization of the virus with the nucleus (DAPI, blue) or lysosome (LAMP1, red) on a per cell basis. Violin plots marked with the median value (dashed lines) and interquartile ranges (dotted lines) for 23 to 106 cells for each of four independent experimental replicates are shown. The difference in mean colocalization of virus with the nucleus and with lysosomes in the presence and absence of HD5 was calculated for each replicate for each genotype. For the C4 vertex and hexon HVR1/C4 vertex chimeras, only data from 6 h p.i. was used. The calculated values for each chimera were then compared to HAdV-5 by ordinary ANOVA with Sidak’s multiple comparisons test. All comparisons are not significant except for hexon HVR1 nuclear colocalization, where p = 0.0014. (E) Representative images of cells at 2 h p.i. (HAdV-5 and hexon HVR1 chimera) or 6 h p.i. (C4 vertex and hexon HVR1/C4 vertex chimera) in the presence of HD5 are shown. In the top panels, nuclear borders from the CellProfiler analysis (cyan) are superimposed on AF488 fluorescence (gray). The merge data includes DAPI (blue), AF488 (green), and LAMP1 (red). Note that the AF488 fluorescence was individually adjusted in these images to account for the differences in brightness between genotypes. Scale bars are 10 μm.

Since the thermostability data do not correlate with infectivity phenotypes, we sought to determine if the mechanism of neutralization seen in protocol 2 agrees with a model whereby HD5 blocks uncoating, alters intracellular trafficking, and prevents the virus from reaching the nucleus. To study the effect of HD5 on intracellular trafficking, we used AF488-labeled viruses and followed protocol 2, binding the virus to cells prior to addition of defensin. At 2 h post-infection (p.i.), samples were stained for LAMP1 to visualize lysosomes and with DAPI to visualize the nucleus, imaged, and analyzed for object-based colocalization. In the absence of HD5, less than 80% of the C4 vertex and hexon HVR1/C4 vertex chimeras reached the nucleus by 2 h p.i. (Fig 8B and 8D), which is in contrast to >90% nuclear colocalization for HAdV-5 and the hexon HVR1 chimera (Fig 8A and 8C). Therefore, the C4 vertex and hexon HVR1/C4 vertex chimeras were also examined at 6 h p.i. to account for the slower kinetics of these viruses compared to HAdV-5, which could be a consequence of their higher inherent stability (Fig 7A).

Overall, subcellular localization correlated with infection phenotype. Consistent with HD5-mediated neutralization in protocol 2 of Fig 4, nuclear colocalization of HAdV-5, the C4 vertex chimera, and the hexon HVR1/C4 vertex chimera were all significantly reduced by HD5 (Fig 8A, 8B and 8D). These genotypes generally appeared as a cluster of peri-nuclear virus in the presence of HD5 (Fig 8E). However, the hexon HVR1 chimera, which is resistant to HD5 (Fig 4C), had little change in nuclear colocalization in the presence of HD5 (Fig 8C). Moreover, virions were evenly distributed across the nucleus even in the presence of HD5 (Fig 8E). The effect of HD5 on lysosomal colocalization was not as extensive for HAdV-5 as in prior studies [11] and varied little between genotypes, likely due to the use of epifluorescence versus confocal microscopy. Nonetheless, the intracellular trafficking of HAdV-5 and the chimeric viruses inhibited by HD5 in protocol 2 (Fig 8 and [14,15]) is perturbed in a manner similar to that of our prior studies of HAdV-5 following protocol 1 [11], resulting in an inability of internalized viruses to reach the nucleus.

## Discussion

In this study, we directly demonstrated that enteric α-defensins can impose selective pressure on non-enveloped viral evolution. We previously postulated that selection may occur during fecal-oral transmission due to the abundant expression and high concentration of enteric α-defensins in the intestinal lumen [16]. This hypothesis is based on the observation that naturally occurring human and mouse AdVs are differentially susceptible to α-defensin antiviral activity and that defensin resistance correlates with AdV species that contain fecal-orally transmitted serotypes [9,16,18,27]. Thus, respiratory HAdV serotypes, which are not exposed during transmission to α-defensins in general and HD5 in particular, remain susceptible, while HAdV serotypes transmitted by the fecal-oral route may have evolved resistance or the capability to co-opt α-defensins to enhance infection. It would also explain the α-defensin resistance of echovirus and reovirus, both enteric pathogens [17]. A prior study used a similar approach to select isolates of HIV-1 resistant to retrocyclin, a θ-defensin [28]. The mutations that appeared in both HAdV-5 and HIV-1 during selection increased the positive charge of the viral structural proteins (hexon and gp41, respectively), likely leading to an overall decrease in defensin binding due to the cationicity of defensins. A similar principle of surface charge modulation contributes to the experimental evolution of bacterial resistance to cationic antimicrobial peptides, which also confers resistance to β-defensins [29]. Collectively, there is now direct evidence that defensins can impose selective pressure on the evolution of a wide range of organisms including not only bacteria and enveloped viruses but also non-enveloped viruses.

Our study provides new insight into the interaction of HD5 with the HAdV capsid. Interestingly, all of the viruses studied herein had similar levels of enhanced epithelial cell binding (Fig 6) despite varied amounts of HD5 bound to their capsids (Fig 5). Defensin-mediated increased cell binding likely occurs by neutralizing the repulsive forces of the electronegative capsid in proximity to the cell membrane. In this regard, charge neutralization by polybrene and other cationic molecules has previously been shown to increase transduction efficiency of HAdV-based vectors [30]. However, the interaction of HD5 with HAdV is not simply charge-dependent, since it requires the hydrophobicity, tertiary structure, and ability to multimerize of HD5 [9,12,15]. Thus, variation in surface charge may account for some of the differential binding of HD5 among genotypes, especially in the case of the hexon mutations that arose during selection. But, the discrete changes introduced in our chimeras indicate that a more complex interaction occurs. For example, the RGD loop of PB in the C4 vertex chimera is 49 amino acid residues shorter than that of WT HAdV-5 and has a calculated net charge of -1 compared to -10 for WT HAdV-5. The change in fiber (GYAR in C4 vertex vs. DTET in HAdV-5) also introduces positive charge, yet the C4 vertex chimera binds ~2.3-fold more HD5 than does HAdV-5. Interestingly, the dose-dependence of enhanced infection (Fig 4E) plateaus at an HD5 concentration lower than that required for maximal binding (Fig 5B), consistent with lower affinity capsid interactions mediating enhanced infection that may saturate prior to complete occupancy of higher affinity interaction sites required for neutralization.

Increased cell binding appears to be the major driver of enhanced infection of epithelial cells. We define enhancement as ≥2-fold higher infection in the presence of HD5 than in the absence of HD5. Consistent with this conclusion, enhancement most often occurs when the virus binds HD5 before binding to the cell (protocol 1 in Figs 3 and 4). Charge-neutralization by HD5 binding may facilitate prototypical receptor interactions of fiber and PB. Alternatively, HD5 could also bridge interactions between the virus and cellular lipids, glycans, or an unidentified HD5-specific receptor, which could be mediated by defensin bound to hexon. Hexon-mediated cell binding facilitated by a host protein has been previously described. Coagulation factors IX and X bridge viral binding to heparan sulfate proteoglycans on hepatocytes [31,32]. Lactoferrin also bridges HAdV-5 interactions to an unknown receptor [33,34]. Interestingly, both HD5 and lactoferrin interact with HVR1 [34]. We also speculate that even in situations where the virus is bound to cells prior to the addition of HD5 (protocol 2), HD5 may promote re-attachment of virions that are bound to a low affinity receptor like sialic acid used by HAdV-64 [35]. This may explain the enhanced infection of HAdV-64 in the presence of 10 μM HD5 in protocol 2 (Fig 4D). An alternative model whereby enhancement occurs through increased internalization or more efficient uncoating cannot be formally excluded but is not supported by any of our studies to date. Because shigella is another gastrointestinal pathogen that appropriates HD5 to facilitate infection [19,20], the ability of enteric pathogens to co-opt defensins to promote adhesion may be common.

Despite increased cell binding and the potential for enhanced infection, many HAdV serotypes are nonetheless neutralized by HD5 upon infection of epithelial cells [9]. A major outcome of our investigation is the identification of a novel determinant of HAdV-5 neutralization by HD5 that fundamentally alters our understanding of the α-defensin antiviral mechanism. Our prior studies supported a model where HD5 binds to the fiber and PB proteins at the vertices of the HAdV-5 capsid and stabilizes their interaction [9]. This action blocks uncoating of the capsid during cell entry and restricts release of the membrane-lytic protein VI, which in turn prevents endosome escape and precludes trafficking of the viral genome to the nucleus [11,14]. This mechanism was supported by structural, biochemical, biophysical, and genetic studies [9,11,12,15,36]. In addition, an HD5-mediated uncoating block for HAdV-5 was directly demonstrated in A549 cells [14]. However, we were unable to account for the extensive HD5 binding to the hexons of HAdV-5 in our cryoEM studies [9]. Our new results indicate that the HVR1 loop of hexon functions in cooperation with the vertex proteins as a previously unidentified determinant of HD5-mediated neutralization, which is further supported by the recent demonstration of neutralization and enhancement of HAdV-based vectors by HD5 in a mouse model [37]. If either HVR1 or both vertex determinants (four residues near the N-terminus of fiber and the C-terminal half of PB) are derived from a resistant serotype, then neutralization does not occur. Rather, these viruses are either resistant to HD5 (e.g., the hexon HVR1 chimera) or enhanced. This suggests that the functions of the capsid determinants are interrelated and that the infection phenotype reflects the additive effects of enhanced cell binding and the potency of subsequent neutralization. However, if the virus is bound to its cellular receptor and co-receptor prior to HD5 addition (protocol 2 in Figs 1C and 4), then HVR1 is the major determinant of neutralization. Thus, the virus-cell interaction functionally replaces the vertex in potentiating HD5 neutralization. Mechanistically, this could occur through receptor-induced conformational changes that lead to exposure of HD5-interacting surfaces in the vertex that are buried in the absence of receptor. Alternatively, the receptor-virus interface could provide a novel target for HD5 binding. However, these interpretations suggest that the virus in protocol 1 either doesn’t experience the conformation induced at 4°C in protocol 2 or transitions through it too rapidly for HD5 to exert a neutralizing effect. Moreover, it is unknown how many vertices are receptor-engaged under the conditions of protocol 2. If only a subset are bound, then blocking uncoating triggered through these vertices may be the key step impeded by HD5 binding [38]. The nature of the HVR1 loop also dictates the Hill slope of the HD5 inhibition curve in protocol 2 (Fig 4), suggesting distinct levels of cooperativity and modes of HD5 binding by capsids differing in HVR1 loops. And, C4 vertex-containing viruses are neutralized at a lower HD5 concentration than those with the HAdV-5 vertex, which may be due to the inherently higher thermostability of the C4 vertex (Fig 7A) or to its higher HD5-binding capacity (Fig 5). Although a minimum amount of HD5 binding to the capsid is required for neutralization, there is not a simple correlation between the degree of neutralization and the amount of HD5 bound. Total HD5 bound appears to reflect additive functions of HVR1 and the vertex. However, neither the HAdV-5 nor HAdV-64 vertex binds HD5 to the same extent as the C4 vertex, suggesting altered binding by the artificial interface in the C4 chimera. We also found that fiber stabilization does not directly correlate with the infection phenotypes of the chimeras and that swapping HVR1 also affected the ability of HD5 to stabilize fiber dissociation. Collectively, these findings suggest that our previous model of vertex stabilization mediated only by HD5 interactions with fiber and PB is incomplete.

A model most consistent with our data is that blocking vertex dissociation through HD5 interactions with fiber/PB is insufficient, and a separate hexon-dependent mechanism, perhaps inter- or intra-hexon or hexon-vertex “cross-linking” by HVR1-HD5 interactions, is also required to prevent uncoating. This would account for HD5 density on all four unique hexon positions in the asymmetric unit of the icosahedral capsid in our prior cryo-EM studies [9]. We cannot formally exclude a model where the capsid determinants act cooperatively to coordinate HD5 binding at the vertex, particularly since the HAdV-5 hexon HVR1 loop is long enough to extend from the peri-pentonal hexons towards fiber. Although this would explain the relatively greater HD5 density on the peripentonal hexons of HAdV-5 [9], in such a model it is harder to rationalize a role for the point mutation that arose in hexon HVR7 in the later rounds of selection or to explain the neutralization of naturally occurring HAdVs from other serotypes that lack long HVR1 loops [9]. Both models are also consistent with the phenotypes of the PB chimeras, where intermediate levels of neutralization result from a subset of the PB changes found in the C4 vertex. Further experimentation and extension of our studies to other HAdV serotypes will be required to resolve these possibilities, which are not mutually exclusive. Nonetheless, our trafficking studies of viruses inhibited by HD5 under both protocol 1 [11] and protocol 2 (Fig 8 and [14,15]) support the conclusion that the outcome of blocking vertex dissociation is to prevent internalized HAdV from escaping the endosomal system and reaching the nucleus.

In summary, our studies generated two major insights. First, we demonstrated that HD5 can act as a selective pressure on the evolution of a non-enveloped virus. Second, we identified hexon as a key contributor to α-defensin interactions with AdV. Thus, we have extensively revised the prior model of HD5-mediated neutralization and established the feasibility of this process to shape viral evolution *in vivo*.

## Materials and methods

### Cell lines

HEK 293 cells overexpressing human β5 integrin (293β5) [9] and A549 cells (ATCC) were maintained in Dulbecco’s modified Eagle’s medium (DMEM) with 10% fetal bovine serum (FBS), penicillin, streptomycin, _L_-glutamine, and non-essential amino acids (complete media).

### HD5

Partially purified (89%) linear peptides (ATCYCRTGRCATRESLSGVCEISGRLYRLCCR) were synthesized (LifeTein, Somerset, NJ), oxidatively folded, and purified by reverse-phase high-pressure liquid chromatography (RP-HPLC) [12]. Fractions containing the correctly folded species were lyophilized, resuspended in deionized water, and quantified by absorbance at 280 nm as described [12]. Purity (>99%) and mass were verified by analytical RP-HPLC and MALDI-TOF mass spectrometry. HD5 was stored at -80°C.

### Viruses

An E1/E3-deleted, replication-defective HAdV-5 vector containing a CMV promoter-driven enhanced green fluorescent protein (eGFP) reporter gene cassette was used as the parent construct for all of the novel chimeras created for these studies, which were generated by recombineering in BACmids [9,39]. The C1 chimera was previously referred to as “PB/GYAR” [9]. Designs of chimeras C2-C14 are depicted in Fig 3. Hexon chimeras were created by replacing either HVR1 (bp 19247 to 19336 in HAdV-5, NCBI: AC_000008.1), HVR7 (bp 20090 to 20203), or both HVR1 and HVR7 in the HAdV-5 *hexon* ORF with the corresponding sequences from HAdV-64 HVR1 (bp 18196 to 18243, GenBank: EF121005.1) and HVR7 (bp 19027 to 19161). The combined hexon HVR1/C4 vertex chimera was created by replacing the HAdV-5 HVR1 with that of HAdV-64 in the C4 chimera. A previously described E3 deleted, replication-competent HAdV-64 virus containing a CMV-eGFP ORF was also used to study the effects of HD5 on infection of a virus with a WT HAdV-64 capsid [40]. The fidelity of all BACmid constructs was verified by Sanger sequencing of the recombineered region and by restriction digest of the entire BACmid. In addition, the BACmids of HAdV-64, chimera C1, and chimera C4 were sequenced in their entirety by whole genome sequencing.

To produce virus, 293β5 cells were transfected with viral genomes released from the BACmids by *Pac* I endonuclease digestion. Following amplification over several passages in 293β5 cells to generate sufficient inoculum, approximately eight to ten T175 flasks of 293β5 cells were infected at a multiplicity of infection of ~3. Upon development of complete cytopathic effect, virus was precipitated from the supernatant in 8% polyethylene glycol (PEG) [41]. Virus was then purified from cell lysates and the PEG precipitate using a CsCl gradient as previously described [14]. Purified virus was dialyzed against three changes of 150 mM NaCl, 40 mM Tris, 10% glycerol, 2 mM MgCl_2_, pH 8.1, snap frozen in liquid nitrogen, and stored at -80°C. The viral particle concentration was determined by Qubit fluorometric quantification (ThermoFisher) against a DNA standard (1 μg = 2.34E+10 virions). Genomic DNA was isolated from purified virus using the GeneJET genomic DNA purification kit (Thermo Fisher), and the fidelity of the changed regions was verified by Sanger sequencing. For biochemical assays, viral protein concentration was determined by Bio-Rad Protein Assay with a bovine serum albumin standard.

### Infection assays

As in our previous studies [15], we employed two protocols, which differed in the order of addition of HD5 to the virus relative to cell binding. All infection assays were performed in black wall, clear bottom 96-well plates seeded with either A549 or 293β5 cells. Protocol 1 (Figs 2,3 and 4): virus and HD5 were incubated together on ice in serum free media (SFM) for 45 min. Cells were then washed twice with SFM to remove any residual serum, the virus/HD5 mixture was added to the cells, and the plate was shifted to 37°C. Protocol 2 (Figs 1C and 4): cells were incubated on ice for 5 min, washed once with cold SFM, and virus in SFM was then added. After incubation for 45 min on ice, the inoculum was removed, the cells were washed once with SFM, and HD5 in SFM was added. After incubation for 45 min on ice, the plate was shifted to 37°C. For both methods, the cells were washed with SFM after 2 h of incubation at 37°C, and the media was replaced with complete media made from phenol red free DMEM. Cells were imaged 20–28 h p.i. on a Typhoon (GE Healthcare) or Sapphire (Azure) imager, and ImageJ was used to quantify background-subtracted total monolayer fluorescence. Data are shown as a percent of control infection in the absence of HD5. Concentrations of each virus were determined in advance that result in 50–80% of maximum signal for inhibition studies or 10–25% of maximum signal for enhancement studies.

### HD5 selection

As previously described, we used purified “mutator” adenovirus (HAdV-5.polF421Y) that was passaged for 10 rounds in 293β5 cells to generate diversity in the starting population [24]. We infected 293β5 cells in 12-well plates under selection with HD5 at the IC_90_ following protocol 2 above. We used a ~6-fold lower MOI for control virus passaged in the absence of HD5 to yield comparable infection levels. Upon development of complete cytopathic effect, which typically occurred 3 to 4 days p.i., the entire culture of cells and media was collected. A clarified freeze/thaw lysate generated from the infected cells was combined with the supernatant and snap frozen in liquid nitrogen for storage at -80°C. An aliquot was used to determine the infectious titer of each sample to ensure a similar level of infection for each round of selection. In addition, the HD5 IC_90_ was determined periodically on 293β5 cells to recalibrate the selective pressure (Fig 1A). The entire process of 50 rounds of selection was not repeated. For subsequent assays, selected viral pools were amplified in the absence of HD5 over ~5 passages in 293β5 cells to generate sufficient inoculum and then purified from preparations of eight to ten T175 flasks of 293β5 cells as described above.

### Plaque purification

Pooled virus from rounds 10, 40, and 50 of the selection were plaque purified on 293β5 cells in a 6-well plate. For the initial infection, purified virus was used to infect cells at low MOI (0.004–0.03), and cultures were overlaid with complete media containing 1% Difco Noble Agar. After 7–14 d, cells and agar plugs from individual plaques were harvested using a pipet tip, resuspended in 100 μL of complete media, and lysed through three freeze-thaw cycles. The plaque-purified isolates were subjected to two additional rounds of plaque purification, expanded, and purified as described above. At intermediate steps, PCR and Sanger sequencing were used to identify plaques containing mutations in the *hexon* and *L4-100K* ORFs.

### Whole genome sequencing

Genomic DNA was extracted and quantified from purified preparations of the plaque purified viruses and the pooled viruses from every 5^th^ round of HD5 selection and the 10^th^ and 20^th^ round of control selection. The Nextera XT DNA Library preparation kit (Illumina) was used to tagment and barcode the genomic DNA. A MiSeq v3 150 cycle reagent kit (Illumina) was used to sequence the libraries with 75 base paired-end reads. The data were analyzed using the BreSeq pipeline to align the sequences to the parent genome and identify mutations [42].

### Virus-defensin binding assay

To measure HD5 binding, 2.5 μg of purified virus was incubated with 5, 10, or 20 μM HD5 in a buffer consisting of 150 mM NaCl, 20 mM Tris, 5% glycerol, 1 mM MgCl_2_ on ice for 45 min. Samples were then layered onto a discontinuous gradient containing 300 μl of 30% Histodenz overlaying 200 μl of 80% Histodenz in 20 mM tris pH 7.4. Gradients were centrifuged using an SW55ti rotor with adaptors (Beckman) at 209,0006 × g (avg.) for 1.5 h at 4°C, and the visible virus band was collected. Samples containing HAdV-5 mixed with HD5 that were not subject to centrifugation were used to generate a standard curve for quantification. All samples were reduced with DTT, heated to 95°C for 5 min, and separated by SDS-PAGE (10–20% tris-tricine gel). The gels were stained with Flamingo fluorescent protein gel stain (Bio-Rad) and imaged on a Sapphire Biomolecular Imager (Azure). Protein bands were quantified using Azure Spot software (Azure). The amount of HD5 in each sample was normalized to protein V and protein VII and quantified against the standard curve using Prism 8.3.0 software (GraphPad).

### Binding of Virus/HD5 to Cells

Viruses were labeled using AF488 carboxylic acid, tetrafluorophenyl ester (Thermo Fisher Scientific) as previously described [11]. Labeling appeared to have no effect (C4 vertex), less than a 2-fold effect (HAdV-5, hexon HVR1, and hexon HVR1/C4 vertex), or a 3-fold (HAdV-64) effect on the infectivity of the viruses on A549 cells. To normalize the amount of each virus used for binding and trafficking studies, we used epifluorescence microscopy to quantify the amount of virus internalized by cells at 2 h p.i. for a dilution series of each virus. Because each virus was labeled to a different level of brightness, the absolute shift in GMF of cells bound to each virus varied.

Similar amounts of AdV5.eGFP, HAdV-64, and the chimeric viruses were incubated with 0, 2.5, 5, and 10 μM HD5 on ice in 60 μl SFM. A549 cells (1.0 x 10^5^ cells/sample) were trypsinized and incubated in suspension on ice in PBS containing 0.2% sodium azide to prevent endocytosis. The virus-defensin complexes were added to the cells (final volume of 100 μl/sample), incubated on ice for 45 min, washed twice with cold PBS to remove unbound virus, and fixed with 1% paraformaldehyde. Samples were analyzed on a BD FACS Canto II, and GMF values for the AF488 signal of the entire cell population were calculated using FlowJo v10.7.1.

### Quantification of subcellular localization and image analysis

To measure the percentage of virus in the nucleus or lysosomes in the presence or absence of HD5, A549 cells were seeded at 3.5 x 10^4^ cells/mL on glass coverslips at least 24 h prior to infection. AF488-labeled AdV5.eGFP and chimeric viruses were diluted in SFM to achieve similar numbers of visible labeled virions/cell (30–100, depending on genotype and replicate), as described above. Virus was bound to cells in 50 μL SFM at 4°C for 1 h in the dark. Unbound virus was removed by two washes of cold SFM, and samples were then incubated with or without 10 μM HD5 in SFM at 4°C in the dark. After 1 h, coverslips were shifted to 37°C and incubated for the amount of time (2 h or 6 h) indicated in Fig 8. Note that samples incubated for a total of 6 h were incubated for 2 h in SFM and for the remaining 4 h in complete media. After incubation, coverslips were washed with PBS, fixed for 15 min in 2% PFA at RT, washed with PBS, and then incubated for 20 min at RT in permeabilization buffer (20 mM glycine, 0.5% Triton-X 100, PBS). Cells were sequentially stained with mouse anti-human CD107a antibody (BD #555798) at 1:200 and with goat anti-mouse IgG AF594 (Thermo Fisher Scientific #A11005) at 1:1000 in antibody staining buffer (1% BSA and 0.05% Tween-80 in PBS) for 1 h at RT. The nucleus was then labeled using 500 ng/mL DAPI for 5 min, and coverslips were mounted with ProLong Gold (Life Technologies #P36930).

Z-series of images spanning the entirety of the cells were obtained by epifluorescence microscopy using a Keyence BZ-X710 microscope in high resolution mode with a 100X objective. A maximum intensity z-profile of each field of view was generated using the CellProfiler version 3.1.9 [43] pipeline in S1 Data. CellProfiler was also used for colocalization analysis using the pipeline in S2 Data. Briefly, DAPI fluorescence was smoothed by a Gaussian filter, and nuclei were identified upon application of thresholding by the Otsu method. Nuclear border propagation guided by fluorescence in the Alex Fluor 594 channel, after application of Otsu thresholding, was used to define cell borders. Speckles were enhanced in the AF488 and AF594 channels, and a robust background thresholding was applied to identify virions and lysosomes, respectively. These objects were then shrunk and expanded to uniform circles with diameters of 2 pixels (virions) and 3 pixels (lysosomes) based on the distribution of dimensions of the raw objects. For quality control, the identified objects were visually inspected and manually adjusted using the pipeline in S3 Data, as needed. Object-based colocalization was then used to identify virions overlapping with lysosomes or the nucleus in each cell. Cells containing fewer than 10 virions were excluded from analysis.

### Thermostability assay

To measure the capsid association of fiber, 250 ng of purified virus was incubated on ice with or without the indicated concentrations of HD5 for 45 min in SFM containing 0.05% BSA, 150 mM NaCl, and 10 mM HEPES pH 7.5. Samples were heated in a thermocycler at the indicated temperatures for 10 min and loaded onto a discontinuous gradient containing 400 μl of 30% Histodenz and 200 μl of 80% Histodenz in 20 mM tris pH 7.4. Samples were centrifuged as described above. Fractions were collected as follows: 90 μl from the top (supernatant), 2 middle fractions of 150 μl, and then 90 μl (virus band). The supernatant and band fractions were reduced with DTT, heated to 95°C for 5 min, separated by SDS-PAGE (12% tris-glycine gel), transferred to nitrocellulose, and probed by immunoblot for fiber using the 4D2 monoclonal antibody (ThermoFisher) and an AF647-conjugated secondary antibody. Blots were imaged and quantified as described above to determine the fraction of total fiber in each sample that was present in the virus band fraction. Prism 8.3.0 was used for non-linear regression analysis to calculate Tm and HD5 concentrations resulting in 50% fiber dissociation in Fig 6.

### Statistical analysis and structural rendering

Statistical analysis was performed using Prism 8.3.0. Specific analyses are indicated in the figure legends. For all tests, not significant (ns), P > 0.05; *, P = 0.01 to 0.05; **, P = 0.001 to 0.01; ***, P = 0.0001 to 0.001; ****, P < 0.0001. Structural figures were generated using the PyMOL Molecular Graphics System, Version 2.0.7 Schrödinger, LLC.

## Supporting information

S1 Data CellProfiler Pipeline 1. This CellProfiler 3.1.9 pipeline creates a maximum intensity z-projection of the image stack from each channel.
(CPPROJ)

S2 Data CellProfiler Pipeline 2. This CellProfiler 3.1.9 pipeline uses the z-projection images from the pipeline in S1 Data and calculates the number of virions per cell that co-localize with the nucleus and with lysosomes. This data is exported as an Excel spreadsheet. Output images include RGB tiffs of the raw fluorescence (“Raw RGB”), of the processed images used for object identification (“RGB”), of the outlines of all of the identified objects (cells, nuclei, viruses, lysosomes; “Outlines”), and of the identified viruses with the raw and processed fluorescence of the green channel (“QCAdV”).
(CPPROJ)

S3 Data CellProfiler Pipeline 3. This CellProfiler 3.1.9 pipeline performs the same analysis as the pipeline in S2 Data; however, it allows for manual editing of the identified objects.
(CPPROJ)
